# Flexural band gaps and vibration control of a periodic railway track

**DOI:** 10.1038/s41598-021-97384-3

**Published:** 2021-09-13

**Authors:** Mohd Iqbal, Anil Kumar, Mahesh Murugan Jaya, Oreste Salvatore Bursi

**Affiliations:** 1 Department of Mechanical and Industrial Engineering, Indian Institute of Technology Roorkee, Roorkee, 247667 India; 2 Department of Structural and Geotechnical Engineering, Politecnico di Torino, 10129 Torino, Italy; 3 Department of Civil, Environmental and Mechanical Engineering, University of Trento, 38123 Trento, Italy

**Keywords:** Engineering, Applied physics

## Abstract

Periodic structures exhibit unique band gap characteristics by virtue of which they behave as vibro-acoustic filters thereby allowing only waves within a certain frequency range to pass through. In this paper, lateral and vertical flexural wave propagation and vibration control of a railway track periodically supported on rigid sleepers using fastenings are studied in depth. The dispersion relations in both lateral and vertical directions are obtained using the Floquet-Bloch theorem and the resulting dispersion curves are verified using finite element models. Afterwards, tuned mass dampers (TMDs) with different mass ratios are designed to control vibrations of the examined rail in both the directions. Moreover, the influence of damping of rail and resonators on band gap characteristics is investigated. As a replacement to the conventional TMD, a novel possibility to control vibration relies on using another existing rail as a lateral distributed resonator (LDR). Although the effectiveness of LDR is lower than that of localized resonators, the former represents a simple and promising way to control vibrations. Efficacy of the proposed control methods is finally verified by applying a random Gaussian white noise input. The study presented here is useful to understand the propagation and attenuation behavior of flexural waves and to develop efficient and novel vibration control strategies for track structures.

## Introduction

Railway system is the most widely used medium of transportation between urban and rural areas. Due to traffic congestion issues in cities, the railway serves as a reliable alternative and is realized as the safest, on time, rapid and most convenient medium of transportation. However, due to the significant increase in speed and the operating frequency of trains, the interaction of wheel/rail is greatly enhanced and results in excessive noise and vibrations in tracks^1^. This can cause fatigue damage, corrugations in rails, loosening of fasteners and cracks in sleepers. A significant part of the railway infrastructure budget is thus required to maintain the safety of such tracks. Also, such excessive vibrations affect both strength and serviceability requirements of buildings adjacent to tracks. At the same time, the generation of excessive noise results in noise pollution which is a major concern to the mental health of residents and can lead to hearing loss. Therefore, it is essential to protect rail track structures from undesired waves and large vibration amplitudes.

**Figure 1:**
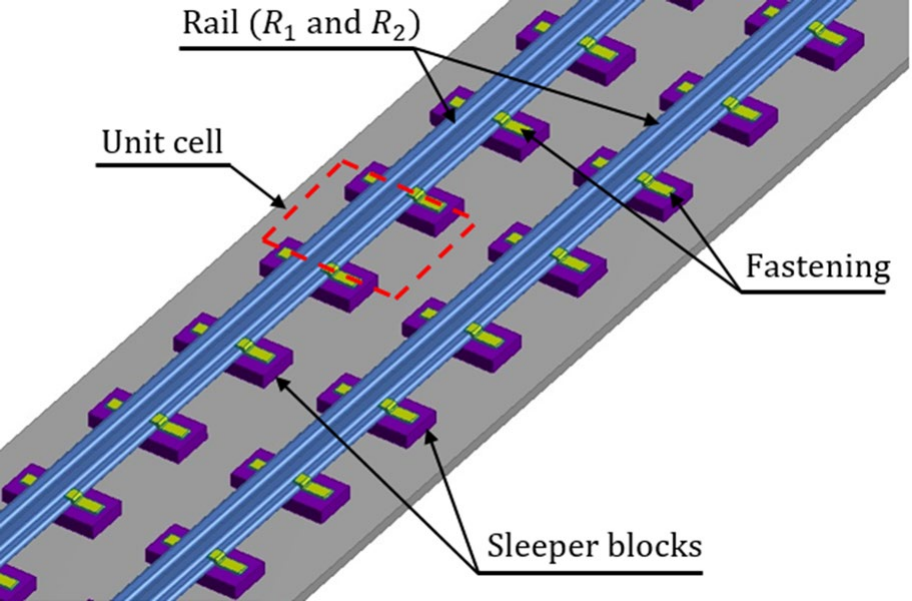
Layout of a ballastless railway track fixed on sleeper blocks using fasteners.

Propagation of elastic waves in periodic structures received much attention of researchers/scientists for decades^2,3^. Meanwhile, the concept of phononic crystals (PCs) introduced from solid-state physics opened a new direction to study the acoustic/elastic wave propagation in periodic structures. Differently from conventional periodic structures, PCs are a new class of materials/structures made by a periodic arrangement of artificial structural units. Such structures possess a unique wave filtering property and thereby exhibit band gaps in certain frequency ranges. This is a result of either the Bragg scattering^2^ or a local resonance^4,5^. The frequency ranges wherein the freely propagating acoustic/elastic waves get attenuated are represented as band gaps or stop bands while waves of the remaining frequencies pass freely creating pass or propagation bands. Earlier studies conducted on band gaps in periodic structures are based on the Bragg scattering mechanism^2,3^. When the characteristic unit cell length $l$ of a periodic structure is comparable to the wavelength $\lambda$ of the waves in the structure, Bragg band gaps are induced. They occur around the frequencies governed by the Bragg condition $l=n\left(\frac{\lambda }{2}\right)$, where $n=\mathrm{1,2},3,\dots$. To date, several studies have been carried out in the context of band gaps in periodic beams^2,6^, piping systems^7,8^, plates^3,9^ and railway tracks^10,11^. However, PCs with locally resonant units are classified as acoustic/elastic metamaterials because of their effective attenuation properties^12^. In addition to Bragg band gaps, locally resonant PCs entail additional band gaps induced by local resonances^4^. Recently, vibration control strategies based on such local resonances were used to filter the propagation of undesired waves in metamaterial beams^13,14^, rods^15^, shafts^16,17^ and piping systems^8^. Moreover, vibration transmission behavior in the vertical and lateral direction of railway tracks like the one depicted in Fig. 1, were investigated by many researchers^18–23^.

In the context of passive control, vibration in a structure can be reduced through different mechanisms. One of the commonly employed methods consists of attaching a secondary unit called tuned mass damper (TMD) to the main structure^24^. TMD consists of a mass that is attached to the main structure using a spring-damper element. The principle of TMD lies in transferring energy from the main system to the secondary system and dissipating it through this secondary system^25,26^. In order to harness an efficient energy dissipation, a TMD has to be optimally designed. For a given mass ratio, this is achieved by adjusting the stiffness and damping values so as to minimize any significant response quantity of the main system, typically displacements. Closed-form expressions exist for the optimal values of such damper parameters in case of an undamped single degree of freedom (SDoF) system subjected to harmonic excitations^25^. Similar optimal values for different types of responses and excitations are also available^27,28^. However, such methods are based on simplified assumptions and are applicable only to simple structures. For more accurate designs and for complex structures, TMDs are designed by means of numerical optimization techniques^29–32^.

In the context of railway tracks, different realizations of TMDs exist^33–37^. Most of these systems employ attaching masses on either side of the rail which are allowed to deform in both the lateral and vertical directions. Damping in such cases may be obtained by hysteresis in the attached damping layers^33–35^. Magnetorheological elastomeric rail dampers endowed with variable stiffness obtained by means of magnetic fields also exist^36^. Energy can also be dissipated in TMDs using impacts/pounding of masses^37^.

In this study, flexural band gaps and vibration control of a ballastless periodic track structure is examined. Along this main vein, flexural wave propagation is both theoretically and numerically investigated. More precisely, two types of flexural waves are studied in the track: (1) lateral wave—Wave #A and (2) vertical wave—Wave #B. The dispersion relation that characterizes wave propagation in the rail is derived using the Floquet-Bloch theory of periodic structures and is subsequently verified by a finite element (FE) model. In this respect, Fig. 1 shows a simplified layout of the track consisting of two rails ${R}_{1}$ and ${R}_{2}$ fixed on sleeper blocks using fasteners having translational and rotational stiffnesses in both lateral and vertical directions. The sleeper blocks are assumed to be rigid and any flexibility of the parts underneath is neglected. For modelling the track, a Euler–Bernoulli beam formulation is used^10,18,20,38–42^. The cross-section of the rail starts to deform significantly in high frequency regime^43^ and in such cases shear deformation is important. However, since in the present work, only low frequency range is considered, the shear deformation is neglected and thus for modelling, a Euler–Bernoulli beam will suffice. Torsional effects may occur in rails owing to the sectional asymmetry, but they are neglected as a first approximation.

**Figure 2:**
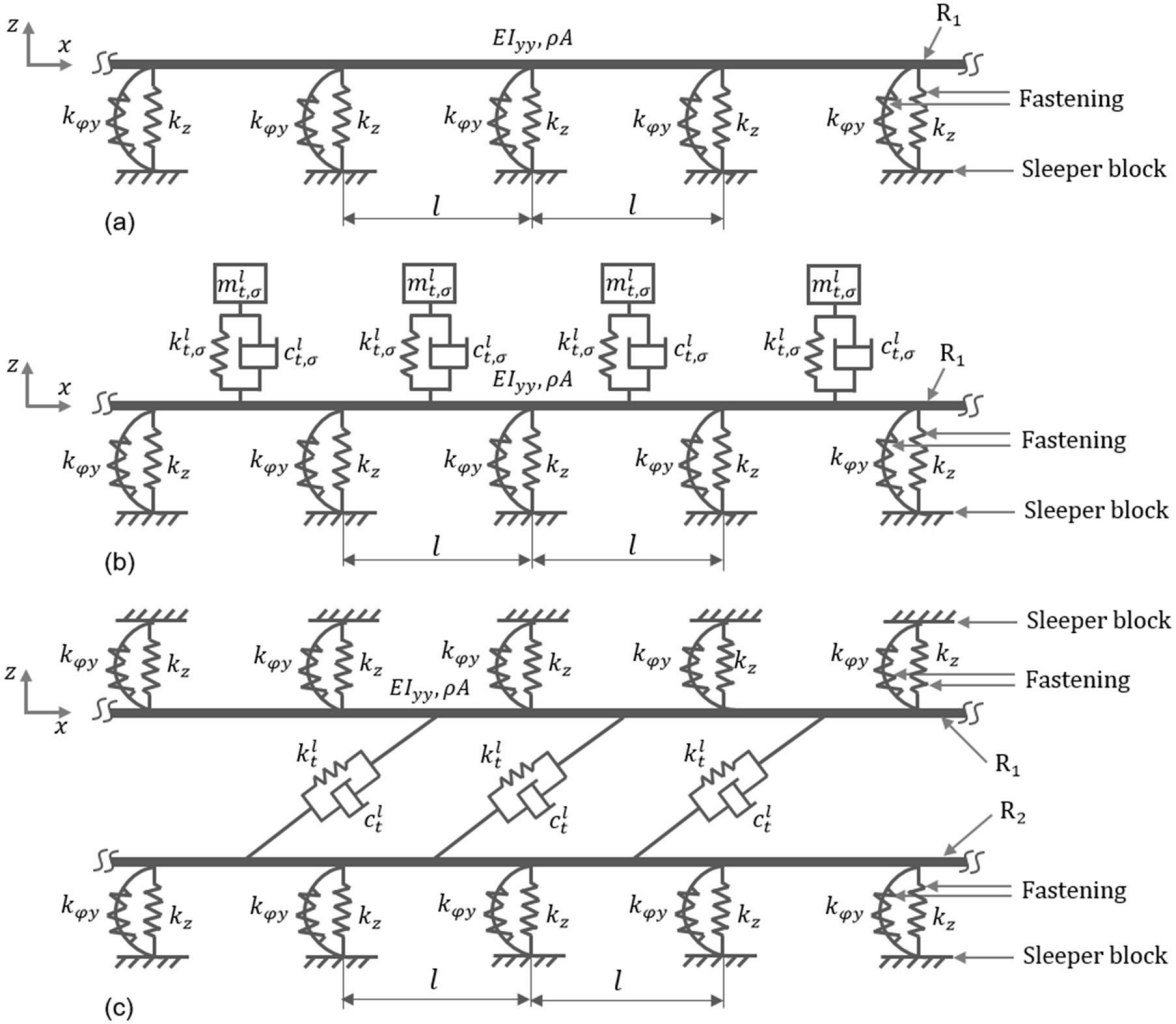
Simplified physical models of a periodic track structure subjected to lateral movement: (**a**) uncontrolled rail ${R}_{1}$; (**b**) rail ${R}_{1}$ endowed with lateral localized resonators (LLRs) in the middle of each unit cell; and (**c**) the adjacent spans of rails ${R}_{1}$ and ${R}_{2}$ connected using spring-damper systems.

In order to tune band gap properties, localized resonators in both lateral (LLRs) and vertical (VLRs) directions are attached at the middle of each unit cell/span of the rail. The configurations of the track in lateral direction (Wave #A) without and with the LLRs are illustrated in Fig. 2a,b, respectively. Identical configurations are also used for the rail in the vertical direction (Wave #B). In this respect, coordinates $x$, $y$ and z, respectively represent the longitudinal, vertical and lateral directions.

A conventional TMD requires the installation of additional mass along with stiffness and damping components. However, in the case of lateral distributed resonator (LDR), any already existing mass in the system is utilized as a part of control mechanism^8^. The concept of LDR can be easily implemented in situations where multiple beam-like structures are present, such as pipe-rack systems, railway tracks, etc. Here, the mass of another rail already existing in the rail track system can be used as a vibration control mechanism. Thus, in the context of Wave #A, a vibration control mechanism is proposed by coupling rails ${R}_{1}$ and ${R}_{2}$ using spring-damper systems as shown in Fig. 2c. This can be practically realized by connecting them using simple springs that are endowed with targeted equivalent stiffness and damping properties. For controlling lateral vibration (Wave #A) in rail ${R}_{1}$, rail ${R}_{2}$ acts as a lateral distributed resonator (LDR) and vice versa. When a train passes over the track, elastic/acoustic waves propagate in both the rails ${R}_{1}$ and ${R}_{2}$ simultaneously. It is assumed herein that Wave #A propagates in both the rails in the same phase. A simple configuration to couple both the rails is to connect their corresponding mid spans using a spring-damper system^8^. Here, this may not be effective as both the rails vibrate with the same amplitude, frequency and phase making the spring-damper system ineffective. Therefore, the two rails are attached as shown in Fig. 2c, i.e., the mid-span of one rail is connected to the middle of the adjacent span of the other rail. Derivation of dispersion relations is not straightforward for the case when LLRs/LDRs are attached to the rail or when damping is considered. Therefore, in these situations, a numerical model is established to conduct the relevant studies. The optimal values of spring and damper parameters of TMDs in both lateral and vertical directions are obtained using a genetic algorithm-based optimization.

The influence of damping of both rail ${R}_{1}$ and resonators on the band gaps is also studied herein. To show the effectiveness of the proposed control methods, the response of uncontrolled ${R}_{1}$ is compared against the different controlled cases. In addition, to evaluate the performance of the optimized LLRs/LDRs and VLRs solutions, a Gaussian white noise load is applied and the resulting response is compared. Results show that in the context of vibration control, LDRs work less efficiently than LLRs; nonetheless, as LDRs do not require any additional mass, they may lead to cost-saving solutions.

It is necessary to examine the propagation and attenuation characteristics of flexural waves in tracks and this warrants the use of the presented analytical dispersion relations. This study also proposes innovative and efficient control strategies that can help to control the undesirable vibration in tracks, thereby increasing their service life. The concepts and methods presented herein are not limited to rails and can be implemented in similar periodically supported structures such as pipelines on rack, bridge supported on multiple piers, etc.

## Methods

### Theoretical modelling and formulation of dispersion relationships

**Figure 3:**
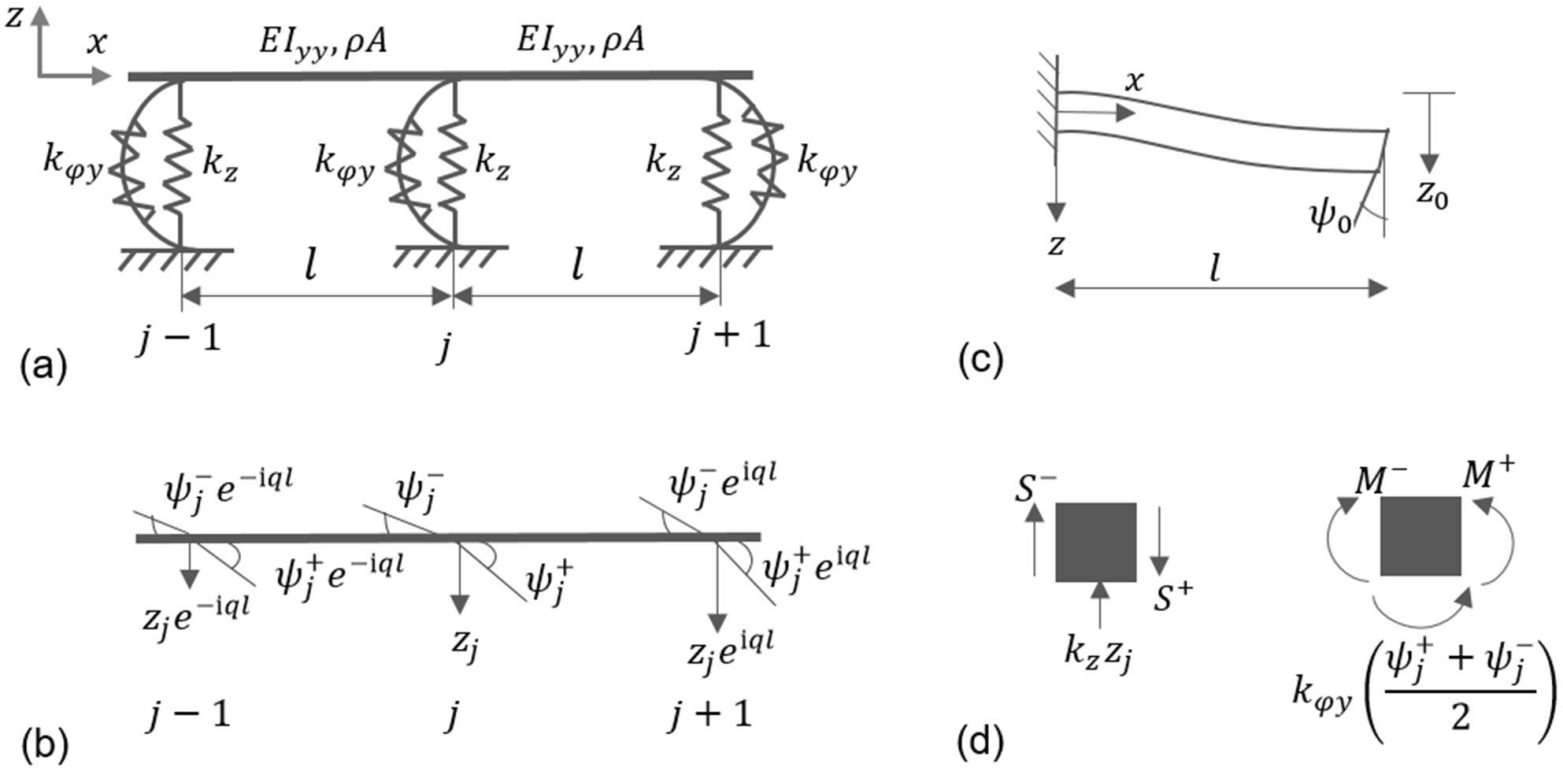
Theoretical modelling of a track structure in the lateral direction. (**a**) Two-unit cells; (**b**) periodic Floquet-Bloch condition imposed to the nodes for angular and transverse displacements; (**c**) single unit cell represented as a simple beam with rotation ${\psi }_{0}$ and transverse displacement ${z}_{0}$ at free end and clamped at the other end; and (**d**) equilibrium of forces and moments at node $j.$

An infinite periodic rail track of span $l$ illustrated in Fig. 2a is adopted to investigate the flexural wave propagation characteristics. The equations are derived for the track in the lateral direction. By changing the parameters, the same relations hold in the vertical direction as well. Each unit cell is composed of a single span of the rail supported on both ends by rigid sleeper blocks connected using fasteners. Two such adjacent unit cells are depicted in Fig. 3a. The governing equation of motion of the undamped rail considered as an Euler- Bernoulli beam is given as,

1 $$\frac{{\partial }^{2}}{\partial {x}^{2}}\left[E{I}_{yy}\frac{{\partial }^{2}z(x,t)}{{\partial x}^{2}}\right]+\rho A\frac{{\partial }^{2}z(x,t)}{{\partial t}^{2}}=0$$

where $E$ and ${I}_{yy}$ are the modulus of elasticity and second moment of inertia about $y$ axis, respectively; $\rho$ and $A$ respectively denote the density and cross-sectional area and $z(x,t)$ represents the transverse displacement as a function of the spatial coordinate $x$ and time $t$.

A steady-state harmonic solution of the form $z\left(x,t\right)=Z(x){e}^{i\omega t}$ is assumed, which when substituted in Eq. (1) yields,

2 $$E{I}_{yy}{Z}^{4}\left(x\right)-\rho A{\omega }^{2}Z\left(x\right)=0$$

where $\omega$ is the angular frequency. The solution of (2) provides the beam displacement amplitude $Z\left(x\right)$ which can be expressed as,

3 $$Z\left(x\right)={D}_{1}\,{\mathrm{cos}}(\alpha x)+{D}_{2}\,{\mathrm{sin}}(\alpha x)+{D}_{3}\,{\mathrm{cosh}}(\alpha x)+{D}_{4}\,{\mathrm{sinh}}(\alpha x)$$

where $\alpha ={\left(\frac{\rho A{\omega }^{2}}{E{I}_{yy}}\right)}^\frac{1}{4}$ denotes the wave number of the flexural wave in the beam.

By applying the Floquet-Bloch periodic condition at each node of the two-unit cells shown in Fig. 3b, transverse displacements of the generic nodes $j+1$ and $j-1$ is related to that at node $j$ as

4 $${z}_{j+1}={z}_{j}{e}^{iql},{z}_{j-1}={z}_{j}{e}^{-iql}$$

where $l$ represents the length of the unit cell, $i$ is $\sqrt{-1}$ and $q$ signifies the Bloch parameter or the wavenumber, which is related to the wavelength $\lambda$ as $\lambda =2\pi /q$. Similar relations are employed for shear forces, bending moments and rotations.

The constants ${D}_{1}$, ${D}_{2}$, ${D}_{3}$ and ${D}_{4}$ in (3) are obtained by means of the boundary conditions illustrated in Fig. 3c, which are used to calculate the bending moments $M$ and shear forces $S$ on both sides of the node $j$. The expressions for dynamic compliance coefficients^44,45^ at $x=0$ and $x=l$ for ${z}_{0}=1$ and ${\psi }_{0}=0$ read,

5 $$\begin{aligned}&{S}_{0}^{^{\prime}}=\frac{{\alpha }^{3}{EI}_{yy \, } [\mathrm{sin}\left(\alpha l\right)+\mathrm{sinh}(\alpha l)]}{1-\mathrm{cos \, } \left(\alpha l\right)\mathrm{cosh \, } (\alpha l)}\\&{S}_{l}^{^{\prime}}=\frac{{\alpha }^{3}{EI}_{yy \, }[\mathrm{cosh \, }\left(\alpha l\right) \mathrm{sin \, } \left(\alpha l\right)+\mathrm{cos \, }(\alpha l)\mathrm{sinh \, } (\alpha l)]}{1-\mathrm{cos \, } \left(\alpha l\right)\mathrm{cosh \, }(\alpha l)}\\ &{M}_{0}^{^{\prime}}=\frac{{\alpha }^{2}{EI}_{yy \, } [\mathrm{cos \, } \left(\alpha l\right)-\mathrm{cosh \, } (\alpha l)]}{1-\mathrm{cos \, } \left(\alpha l\right)\mathrm{cosh \, } (\alpha l)}\\&{M}_{l}^{^{\prime}}=\frac{{\alpha }^{2}{EI}_{yy \, } [\mathrm{sinh \, } (\alpha l)\mathrm{sin \, } \left(\alpha l\right)]}{1-\mathrm{cos \, } \left(\alpha l\right)\mathrm{cosh \, } (\alpha l)}\end{aligned}$$

For ${z}_{0}=0$ and ${\psi }_{0}=1$, the dynamic compliance coefficients are given by

6 $$\begin{aligned}&{S}_{0}^{\prime\prime}=\frac{{-\alpha }^{2}{EI}_{yy}[\mathrm{cosh}\left(\alpha l\right)-\mathrm{cos}(\alpha l)]}{1-\mathrm{cos}\left(\alpha l\right)\mathrm{cosh}(\alpha l)}\\ &{S}_{l}^{\prime\prime}=\frac{-{\alpha }^{2}{EI}_{yy}\left[\mathrm{sinh}\left(\alpha l\right)\mathrm{sin}\left(\alpha l\right)\right]}{1-\mathrm{cos}\left(\alpha l\right)\mathrm{cosh}\left(\alpha l\right)}\\ & {M}_{0}^{\prime\prime}=\frac{-\alpha {EI}_{yy}[\mathrm{sin}\left(\alpha l\right)-\mathrm{sinh}(\alpha l)]}{1-\mathrm{cos}\left(\alpha l\right)\mathrm{cosh}(\alpha l)}\\ &{ M}_{l}^{\prime\prime}=\frac{-\alpha {EI}_{yy}[\mathrm{cosh}\left(\alpha l\right) \mathrm{sin}\left(\alpha l\right)-\mathrm{cos}(\alpha l)\mathrm{sinh}(\alpha l)]}{1-\mathrm{cos}\left(\alpha l\right)\mathrm{cosh}(\alpha l)}\end{aligned}$$

With reference to Fig. 3b, the shear forces and bending moments at node $j$ are expressed as

7 $$\begin{aligned}&{S}^{-}={-S}_{0}^{^{\prime}}{z}_{j}{e}^{-iql}+{S}_{l}^{^{\prime}}{z}_{j}+{S}_{0}^{\prime\prime}{\psi }_{j}^{+}{e}^{-iql}+{S}_{l}^{\prime\prime}{\psi }_{j}^{-}\\ &{S}^{+}={S}_{0}^{^{\prime}}{z}_{j}{e}^{iql}-{S}_{l}^{^{\prime}}{z}_{j}+{S}_{0}^{\prime\prime}{\psi }_{j}^{-}{e}^{iql}+{S}_{l}^{\prime\prime}{\psi }_{j}^{+}\\&{M}^{-}={M}_{0}^{^{\prime}}{z}_{j}{e}^{-iql}+{M}_{l}^{^{\prime}}{z}_{j}-{M}_{0}^{\prime\prime}{\psi }_{j}^{+}{e}^{-iql}+{M}_{l}^{\prime\prime}{\psi }_{j}^{-}\\&{M}^{+}={M}_{0}^{^{\prime}}{z}_{j}{e}^{iql}+{M}_{l}^{^{\prime}}{z}_{j}+{M}_{0}^{\prime\prime}{\psi }_{j}^{-}{e}^{iql}-{M}_{l}^{\prime\prime}{\psi }_{j}^{+}\end{aligned}$$

The equilibrium of forces and moments at node $j$ in Fig. 3d entails,

$${S}^{+}={S}^{-}+{k}_{z}{z}_{j}$$

8 $${M}^{+}={M}^{-}-{k}_{\varphi y}\left(\frac{{\psi }_{j}^{-}+{\psi }_{j}^{+}}{2}\right)$$

where ${k}_{z}$ and ${k}_{\varphi y}$ represent the translational and rotational stiffness of the fastening, respectively.

The kinematic compatibility condition at node $j$ is given as,

9 $${\psi }_{j}^{-}={\psi }_{j}^{+}$$

Equations (7)-(9) yield a set of linear homogeneous equations in term of ${\psi }_{j}^{-}$, ${\psi }_{j}^{+}$ and ${z}_{j}$ as,

10 $${\psi }_{j}^{-}-{\psi }_{j}^{+}=0$$

11 $$\left({S}_{0}^{\prime\prime}{e}^{iql}-{S}_{l}^{\prime\prime}\right){\psi }_{j}^{-}+\left({{S}_{l}^{\prime\prime}-S}_{0}^{\prime\prime}{e}^{-iql}\right){\psi }_{j}^{+}+\left[2{S}_{0}^{^{\prime}}\mathrm{cos}\left(ql\right)-2{S}_{l}^{^{\prime}}-{k}_{z}\right]{z}_{j}=0$$

12 $$\left({M}_{0}^{\prime\prime}{e}^{iql}-{M}_{l}^{\prime\prime}+\frac{{k}_{\varphi y}}{2}\right){\psi }_{j}^{-}+\left({M}_{0}^{\prime\prime}{e}^{-iql}-{M}_{l}^{\prime\prime}+\frac{{k}_{\varphi y}}{2}\right){\psi }_{j}^{+}+\left[2{iM}_{0}^{^{\prime}}\mathrm{sin}\left(ql\right)\right]{z}_{j}=0$$

Successively, Eqs. (10)–(12) can be written in a matrix form as,

13 $$\left[\begin{array}{ccc}1& -1& 0\\ {S}_{0}^{\prime\prime}{e}^{iql}-{S}_{l}^{\prime\prime}& {{S}_{l}^{\prime\prime}-S}_{0}^{\prime\prime}{e}^{-iql}& 2{S}_{0}^{^{\prime}}\mathrm{cos}\left(ql\right)-2{S}_{l}^{^{\prime}}-{k}_{z}\\ {M}_{0}^{\prime\prime}{e}^{iql}-{M}_{l}^{\prime\prime}+\frac{{k}_{\varphi y}}{2}& {M}_{0}^{\prime\prime}{e}^{-iql}-{M}_{l}^{\prime\prime}+\frac{{k}_{\varphi y}}{2}& 2{iM}_{0}^{^{\prime}}\mathrm{sin}\left(ql\right)\end{array}\right]\left\{\begin{array}{c}{\psi }_{j}^{-}\\ {\psi }_{j}^{+}\\ {z}_{j}\end{array}\right\}=0$$

A non-trivial solution of (13) entails,

14 $$\left|\begin{array}{ccc}1& -1& 0\\ {S}_{0}^{\prime\prime}{e}^{iql}-{S}_{l}^{\prime\prime}& {{S}_{l}^{\prime\prime}-S}_{0}^{\prime\prime}{e}^{-iql}& 2{S}_{0}^{^{\prime}}\mathrm{cos}\left(ql\right)-2{S}_{l}^{^{\prime}}-{k}_{z}\\ {M}_{0}^{\prime\prime}{e}^{iql}-{M}_{l}^{\prime\prime}+\frac{{k}_{\varphi y}}{2}& {M}_{0}^{\prime\prime}{e}^{-iql}-{M}_{l}^{\prime\prime}+\frac{{k}_{\varphi y}}{2}& 2{iM}_{0}^{^{\prime}}\mathrm{sin}\left(ql\right)\end{array}\right|=0$$

The subsequent solution of (14) provides the dispersion relation of the periodic track structure as,

15 $$\left[4{M}_{0}^{^{\prime}} {S}_{0}^{\prime\prime}{\mathrm{sin}}^{2}\left(ql\right)\right]+\left[2{S}_{0}^{^{\prime}}\mathrm{cos}\left(ql\right)-2{S}_{l}^{^{\prime}}-{k}_{z}\right]\left[{2M}_{0}^{\prime\prime}\mathrm{cos}\left(ql\right)-{2M}_{l}^{\prime\prime}+{k}_{\varphi y}\right]=0$$

The dispersion relation is derived for the rail in the lateral direction, i.e. Wave #A; by replacing the corresponding stiffness values, the same relation holds for the track in the vertical direction, i.e. Wave #B.

### FE modeling

In order to verify the flexural wave propagation behavior obtained based on the dispersion relation (15), a FE model of ${R}_{1}$ placed on rigid sleeper blocks using fasteners is developed employing 2-noded Euler–Bernoulli beam element (BEAM4) available in the ANSYS 2020 R2 software^46^. In the present study, the optimal mesh size for the FE analysis is governed by the range of frequency, and thus the modes considered. The optimal mesh size is decided based on a sensitivity study of the vibration transmittance (Eq. (16)) calculated over the frequency of interest and the computational effort. Based on these two factors, it is decided to discretize each span into ten elements.

If sufficient number of unit cells are used, a finite structure can replicate the band gap characteristics of the corresponding infinite structure. Since in FE modelling, an infinite number of unit cells (spans) cannot be considered, a finite structure composed of 30 unit cells is used to verify the dispersion relations. In order to replicate the flexural wave propagation, a harmonic rotation of the form ${\psi }_{i/p }{e}^{i2\pi ft}$ with $f=\omega /2\pi$ is imposed to the left end of ${R}_{1}$(in the first span). These waves propagate through the rail and the output rotation ${\psi }_{o/p}(f)$ is measured at the right end (last span). For generating the lateral and vertical flexural wave, the input rotation ${\psi }_{i/p }{e}^{{i}2\pi ft}$ is separately applied about the $y$ and $z$ axis. The vibration transmittance ${T}_{\psi } (dB)$ for this system is defined as

16 $${T}_{\psi }=20 {log}_{10}\left|\frac{{\psi }_{o/p}(f) }{{\psi }_{i/p}(f)}\right|$$

### Design of vibration control mechanism

To control the flexural waves in the track for both lateral and vertical cases, a certain frequency range of the first pass band is considered. For an efficient control, both optimal stiffness and damping properties needs to be employed for the controller. For a LLR with a given mass ratio $\upsigma , {\mathrm{where}}\; \sigma ={m}_{t,\sigma }^{l}/{\left(\rho Al\right)}_{{R}_{1}}$ and ${m}_{t,\sigma }^{l}$ is the mass of the resonator/TMD, the optimal stiffness ${k}_{t,\sigma }^{l}$ and damping ${c}_{t,\sigma }^{l}$ determine the efficiency of control strategy. Let ${\Vert {H}_{Control}\Vert }_{\infty }$ and ${\Vert {H}_{Uncontrol}\Vert }_{\infty }$ refer to the peak value of ${\psi }_{o/p}(f)$ with and without LLR, respectively. As a measure of the efficiency of LLR, a performance metric $\eta = {\Vert {H}_{Control}\Vert }_{\infty } /{\Vert {H}_{Uncontrol}\Vert }_{\infty }$ is adopted, which is then minimized to obtain the optimal ${k}_{t,\sigma }^{l}$ and ${c}_{t,\sigma }^{l}$. A lower value of $\eta$ denotes better vibration suppression capabilities of the controlled structure. A genetic algorithm (GA)-based optimization is adopted for the design of a given mass ratio $\sigma$ as follows,

17 $$\left\{{k}_{t,\sigma }^{l}\,{\mathrm{and}}\,{c}_{t,\sigma }^{l} \right\}={\mathrm{argmin}}\left(\eta \right)$$

subjected to,

18 $$\left\{LB\right\} \le \left\{{k}_{t,\sigma }^{l}\,{\mathrm{and}}\,{c}_{t,\sigma }^{l} \right\} \le \left\{UB\right\}$$

$\left\{UB\right\}$ and $\left\{LB\right\}$ represent the upper and lower bound for ${k}_{t,\sigma }^{l}$ and ${c}_{t,\sigma }^{l}$, respectively. The values of these two bounds are selected such that the design variables do not adopt unrealistic values and results in faster optimization. $\eta$ is calculated over the frequency range to be controlled. A similar GA-based optimization is used to determine the optimal spring-damper parameters ${k}_{t,\sigma }^{v}$ and ${c}_{t,\sigma }^{v}$ of the vertical localized resonator (VLR). Finally, the optimal parameters ${k}_{t}^{l}$ and ${c}_{t}^{l}$ for the LDR in the lateral direction are also obtained using a similar optimization.

**Figure 4:**
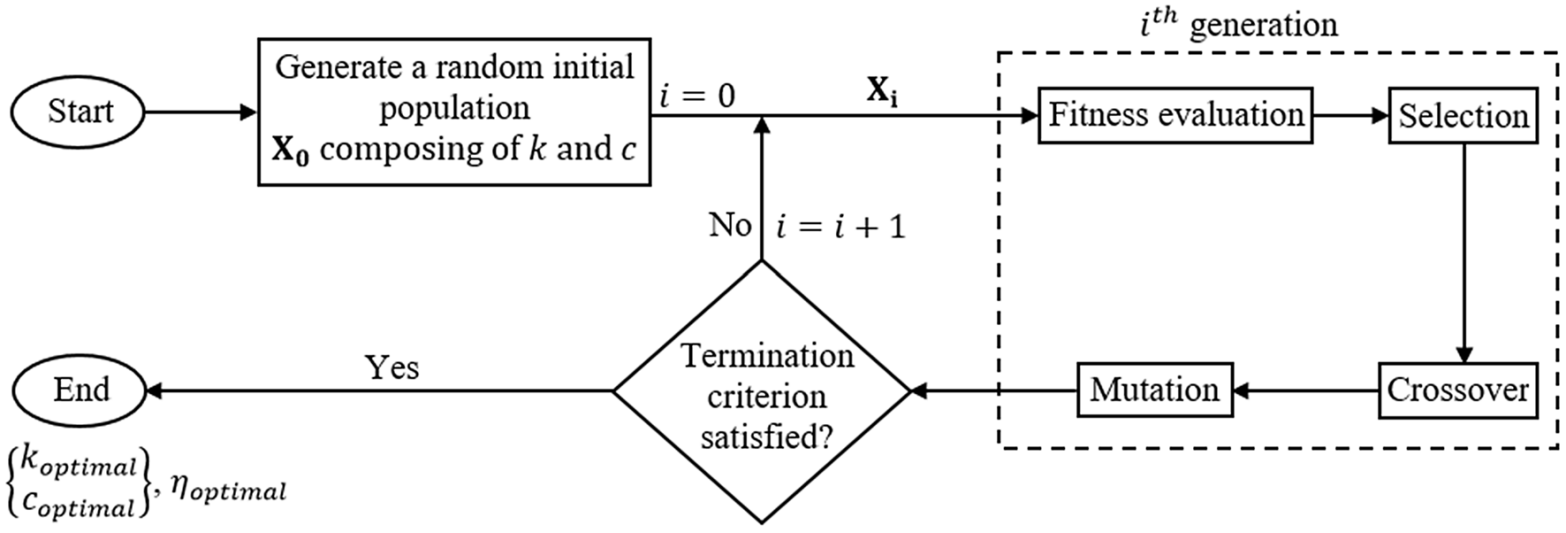
Flow chart of genetic algorithm-based optimization for control mechanism.

In view of the design of TMDs, GA-based methods are widely used^29,30,32^. The concept of GA which is a population-based stochastic search method is based on the principles of natural selection and genetics^47,48^. The optimization starts by selecting a random set of possible initial configurations ${\mathrm{X}}_{0}$ which evolves towards the optimal solution in each generation. A simplified layout of the algorithm is shown in Fig. 4. From any generation $\mathrm{i}$, the ${\mathrm{i+1}}^{th}$ generation is obtained by means of selection, crossover and mutation. Selection involves finding a set of solutions from ${\mathrm{X}}_{\mathrm{i}}$ which has the best fitness values and they are included directly in the next generation. While crossover involves finding new solutions by combining two best solutions from ${\mathrm{X}}_{\mathrm{i}}$, mutation generates new solutions by applying random changes to the solutions in ${\mathrm{X}}_{\mathrm{i}}$. This process is repeated until some desired convergence criterion is satisfied^49^.

## Results

Based on the dispersion relation (15), the propagation characteristics of both Wave #A and Wave #B are initially studied. On the basis of these results, vibration control mechanisms are designed for both the lateral and vertical cases. The influence of LLR/VLR and damping on the band gaps is also determined. Finally, the effectiveness of the optimized LLRs/LDRs and VLRs is verified by imposing a random Gaussian white noise excitation as input.

### Propagation of Wave #A and Wave #B in ${{\varvec{R}}}_{1}$

In order to determine the propagation characteristics of Wave #A and Wave #B, the track shown in Fig. 2a is considered. The dispersion relation provided in (15) corresponds to Wave #A in the undamped rail ${R}_{1}$. By substituting the compliance coefficients relations, the dispersion relation is obtained as,

19 $${[\left\{\mathrm{cosh}\left(\alpha l\right)-\mathrm{cos}\left(\alpha l\right)\right\}}^{2}]{\mathrm{sin}}^{2}\left(ql\right)+\left[\mathrm{cos}\left(ql\right)\{\mathrm{sin}\left(\alpha l\right)+\mathrm{sinh}\left(\alpha l\right)\}-\{\left(\mathrm{cosh}\left(\alpha l\right)\mathrm{sin}\left(\alpha l\right)+\mathrm{cos}\left(\alpha l\right)\mathrm{sinh}\left(\alpha l\right)\right\}-{k}_{z}\left\{\frac{1-\mathrm{cos}(\alpha l)\mathrm{cosh}(\alpha l)}{2{\alpha }^{3}{EI}_{yy}}\right\}\right] \left[\mathrm{cos}\left(ql\right)\left\{\mathrm{sinh}\left(\alpha l\right)-\mathrm{sin}\left(\alpha l\right)\right\}-\left\{\mathrm{cos}\left(\alpha l\right)\mathrm{Sinh}\left(\alpha l\right)-\mathrm{cosh}\left(\alpha l\right)\mathrm{sin}\left(\alpha l\right)\right\}+{k}_{\varphi y}\left\{\frac{1-\mathrm{cos}(\alpha l)\mathrm{cosh}(\alpha l)}{2\alpha {EI}_{yy}}\right\}\right]=0$$

The solution of Eq. (19) yields two pairs of $q$ for each $\omega$: $\pm {q}_{1}$ and $\pm {q}_{2}$; the two signs indicate the same waves propagating in opposite directions. The real part of $(ql)$ represents the phase difference between two adjacent cells while the imaginary part shows the decay rate of the amplitude.

Based on the characteristics of $q$, three types of waves exist. For purely real $q$ ($\mathrm{Im}\left(ql\right)=0$), the waves of all frequencies travel freely through each unit cell thereby giving only pass bands in the dispersion relationships. Here adjacent cells vibrate in phase. Conversely, for purely imaginary $q$ ($\mathrm{Im}(ql)\ne 0$ and $\mathrm{Re}\left(ql\right)=0$ or $\pi$), the amplitude of wave reduces at each unit cell and they are referred to as evanescent waves. Now the adjacent unit cells vibrate either in or out of phase. For a complex $q$, both $\mathrm{Im}\left(ql\right)$ and $\mathrm{Re}\left(ql\right)$ will be non-vanishing, ($\left|\mathrm{Im}(ql)\right|>0$ and $0<\left|\mathrm{ Re}(ql)\right|<\pi$) and the waves propagate and attenuate in the adjacent unit cells resulting in both pass and stop bands in the dispersion curves.

**Figure 5:**
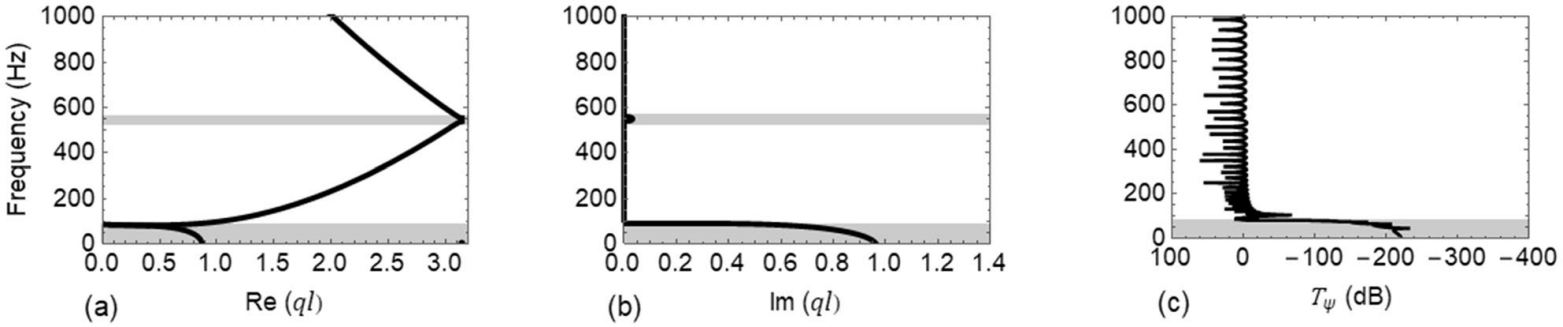
Dispersion curves and transmittance ${T}_{\psi }\left(\mathrm{dB}\right)$ of Wave #A: (**a**) real part of the dispersion relation, Re $(ql)$; (**b**) imaginary part of the dispersion relation, Im $(ql)$; and (**c**) ${T}_{\psi }\left(\mathrm{dB}\right)$.

The properties of the laterally fastened track structure based on Table 1 are used in (19) to obtain the dispersion curves. Dispersion characteristics of only the first wave ($i.e.,{+q}_{1}$) travelling along the positive $x$ direction in the track are investigated. The variation of $\mathrm{Re}\left(ql\right)$ and $\mathrm{Im}(ql)$ with the wave frequency $f=\omega /2\pi$ are plotted in Fig. 5a,b. In the frequency range $\left[0-1000\right] Hz$, two band gaps are found with the frequency ranges of $\left[0-82\right] Hz$ and $\left[541-556\right] Hz$, respectively, and are shown by shaded areas while remaining frequency regions indicate pass bands. To verify these results, an undamped FE model of the rail track consisting of 30 spans is considered. ${T}_{\psi }\left(\mathrm{dB}\right)$ (from Eq. (16)) versus $f$ is plotted in Fig. 5c which shows a perfect correspondence with the first band gap. However, FE model is not able to capture the second band gap. This may be due to its narrow bandwidth and low attenuation of waves.

**Table 1:** Properties of the rail track fastening system^50^.

Component	Property		Value
Rail	Density $(\rho )$		7850 ${\mathrm{kg}/\mathrm{m}}^{3}$
	Modulus of elasticity $(E)$		2.1E11 $\mathrm{N}/{\mathrm{m}}^{2}$
	Area of cross-section $(A)$		77.45E-4 ${\mathrm{m}}^{2}$
	Second moment of inertia	${I}_{yy}$	5.24E-6 ${\mathrm{m}}^{4}$
		${I}_{zz}$	32.17E-6 ${\mathrm{m}}^{4}$
Fastening	Stiffness in lateral direction	Translational $({k}_{z})$	10E6 $\mathrm{N}/\mathrm{m}$
		Rotational $({k}_{\varphi y})$	7E4 $\mathrm{Nm}/\mathrm{rad}$
	Stiffness in vertical direction	Translational $({k}_{y})$	35E6 $\mathrm{N}/\mathrm{m}$
		Rotational $({k}_{\varphi z})$	5E6 $\mathrm{Nm}/\mathrm{rad}$
	Spacing $(l)$		0.625 $\mathrm{m}$

**Figure 6:**
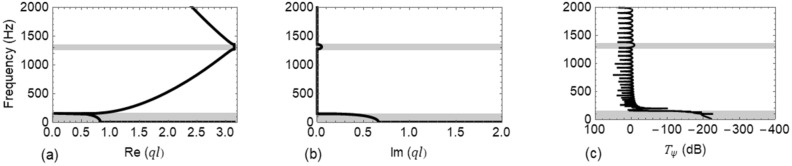
Dispersion curves and transmittance ${T}_{\psi }\left(\mathrm{dB}\right)$ of Wave #B: (**a**) real part of the dispersion relation, Re $(ql)$; (**b**) imaginary part of the dispersion relation, Im $(ql)$; and (**c**) ${T}_{\psi }\left(\mathrm{dB}\right)$.

Similarly, the propagation characteristics of Wave #B is investigated. The properties of the vertically fastened track collected in Table 1 are used with the dispersion relation (19). The vertical direction being stiffer than the lateral entails a higher frequency range from $\left[0-2000\right] Hz$, to be considered. Two band gaps $\left[0-153\right] Hz$ and $\left[1276-1359\right] Hz$ are found in this range and are represented by shaded areas in Fig. 6. Figure 6c corresponds to the FE results and shows an excellent agreement with Fig. 6a,b, respectively.

In Figs. 5 and 6, the first band gap is due to local resonance of rail-fastening system while the second is caused by Bragg scattering^40^. The rail fastening system can be idealized as a single degree of freedom spring-mass system whose stiffness and mass properties respectively correspond to the translational stiffness *(*${k}_{z}=10E6 \,\mathrm{N}/\mathrm{m}$ and ${k}_{y}=35E6\, \mathrm{N}/\mathrm{m}$ for lateral and vertical direction, respectively) of the fastener and the mass of half the span of rail on either side of fastener. The ending frequency of first band gap in both lateral and vertical direction coincide with the natural frequency of the corresponding spring-mass system*.* Here, the Bragg band gap is induced around the frequency governed by the Bragg condition while its starting and ending frequencies depends upon the translational and rotational stiffness of the fasteners.

### Vibration transmission characteristics of a controlled periodic track structure

From the results depicted in both Figs. 5 and 6, it is evident that the bandwidth of the first and second band gap is narrow and the latter also has very low attenuation. Consequently, for a wide frequency range waves can freely pass through the track, causing excessive noise and vibration. Thus, for both the lateral and vertical cases, it is aimed to control a certain frequency range of the first pass band. The full pass band is not considered as the frequency bounds are very large to be efficiently controlled using a SDoF TMD. Thus, the LLRs and VLRs are optimized in the frequency ranges $\left[300-500\right] Hz$ and $\left[500-1000\right] Hz$, respectively.

Along this main vein, identical LLR and VLR are respectively attached to the center of each span of the rail. Figure 2b shows the rail ${R}_{1}$ endowed with LLRs. A similar configuration is adopted with VLRs. For a given mass ratio $\sigma$, the optimal damping coefficient and stiffness are obtained as ${c}_{t,\sigma }^{l}$ and ${k}_{t,\sigma }^{l}$ for the LLR and as ${k}_{t,\sigma }^{v}$ and ${c}_{t,\sigma }^{v}$ for the VLR using (17) and (18). The optimal parameters and the corresponding performance metric $\eta$ calculated for different values of $\sigma$ are listed in Table 2. In the calculation of optimal parameters, material damping ($\xi$) of 2% is used for the rails.

**Table 2:** Optimal TMD parameters for different mass ratios to control lateral and vertical flexural vibrations.

Mass ratio ($\sigma$)	LLR			VLR		
	${k}_{t,\sigma }^{l}$(N/m)	${c}_{t,\sigma }^{l}$(Ns/m)	$\eta$	${k}_{t,\sigma }^{v}$(N/m)	${c}_{t,\sigma }^{v}$(Ns/m)	$\eta$
0.10	1.89E7	5.15E3	0.121	5.24E7	1.10E4	0.199
0.15	2.93E7	7.41E3	0.057	7.56E7	1.68E4	0.106
0.20	4.00E7	9.90E3	0.027	1.03E8	2.07E4	0.058
0.25	5.13E7	1.15E4	0.013	1.24E8	2.61E4	0.034

**Figure 7:**
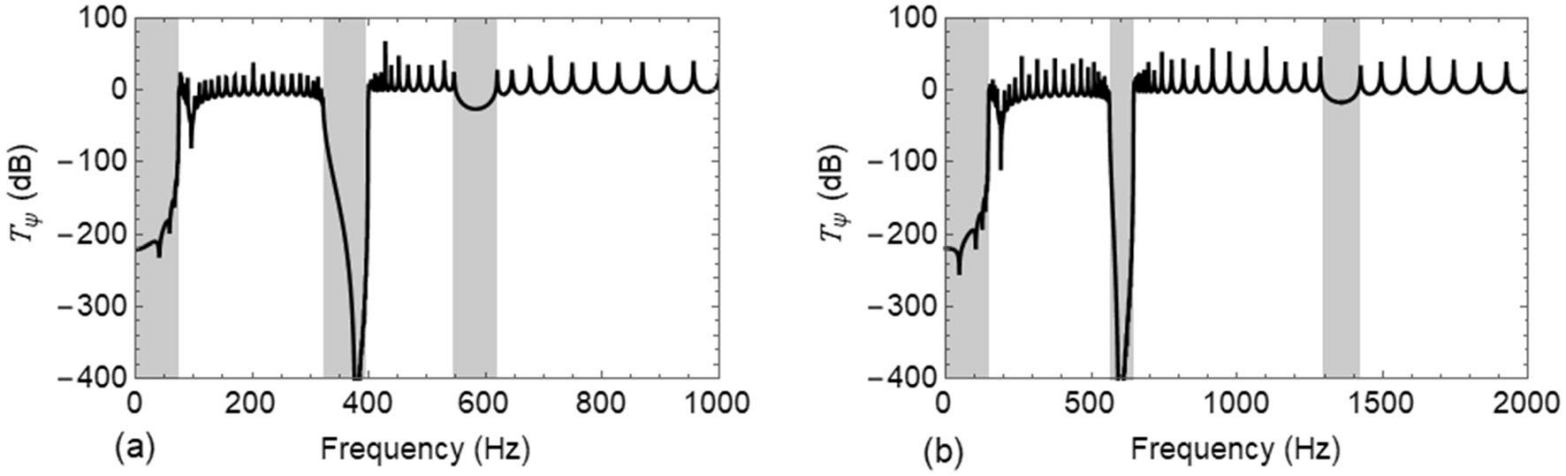
Transmittance ${T}_{\psi }\left(\mathrm{dB}\right)$ of the undamped ${R}_{1}$ when attached with undamped resonators: (**a**) use of lateral localized resonators (LLRs) for Wave #A; and (**b**) use of vertical localized resonators (VLRs) for Wave #B.

Figure 7a,b show the vibration transmittance ${T}_{\psi }$ when damping in both the rail and the resonators are neglected for LLR and VLR, respectively. These plots correspond to a mass ratio $\sigma =0.20$ while the other mass ratios having identical variation of ${T}_{\psi }$ are not reported. In the absence of damping, a new band gap is opened around the natural frequency of the resonator and thus three band gaps are obtained in the frequency response of both the wave types. In the case of Wave #A, the band gap frequency ranges are $\left[0-75\right] Hz$, $\left[318-398\right] Hz$ and $\left[549-618\right] Hz$; conversely, for Wave #B, they are $\left[0-147\right] Hz$, $\left[556-646\right] Hz$ and $\left[1295-1416\right] Hz$. Both are represented by shaded areas in Fig. 7a,b.

**Figure 8:**
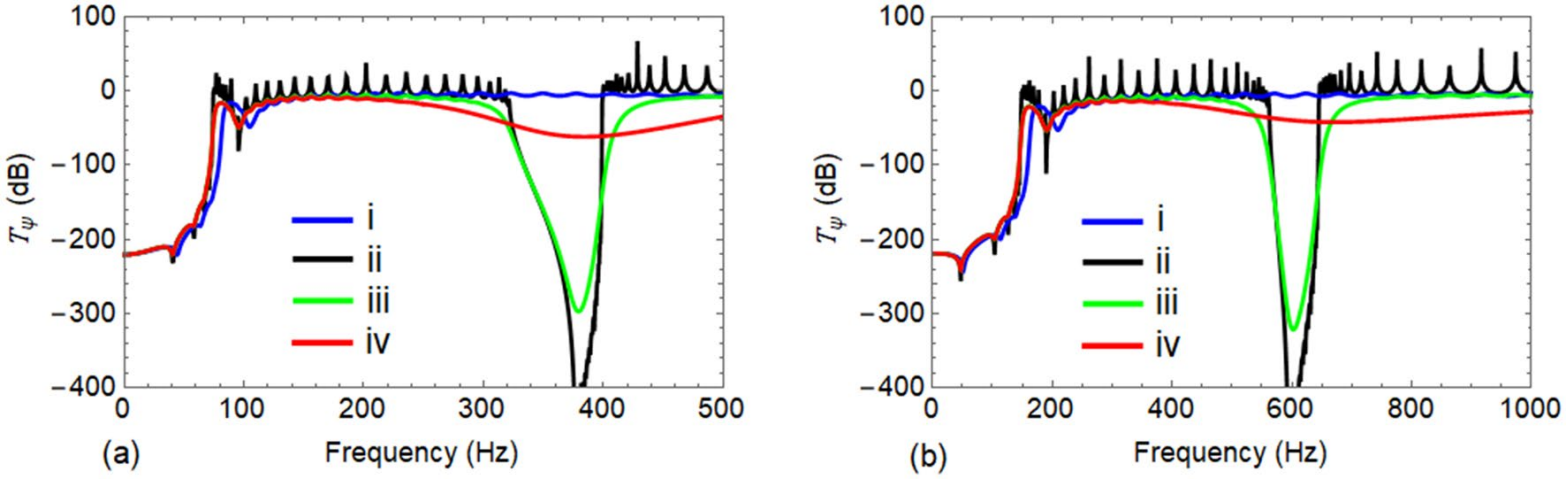
Effect of different damping values on the transmittance ${T}_{\psi }\left(\mathrm{dB}\right)$ of ${R}_{1}$ when attached with LLRs and VLRs, respectively: (**a**) propagation of Wave #A; and (**b**) propagation of Wave #B.

In order to verify the influence of damping of ${R}_{1}$ and the resonators on the band gaps, four cases are examined for both types of wave; (i) uncontrolled ${R}_{1}$ with material damping $(\xi =0.02)$, (ii) both material damping in ${R}_{1}$ $(\xi =0)$ and damping in LLR/VLR is neglected (${k}_{t,\sigma }^{l}=4.0E7, {c}_{t,\sigma }^{l}=0$ for Wave #A and ${k}_{t,\sigma }^{v}=1.03E8, {c}_{t,\sigma }^{v}=0$ for Wave #B), (iii) material damping $(\xi =0.02)$ is considered in ${R}_{1}$ while damping in LLR/VLR is neglected (${k}_{t,\sigma }^{l}=4.0E7, {c}_{t,\sigma }^{l}=0$ for Wave #A and ${k}_{t,\sigma }^{v}=1.03E8, {c}_{t,\sigma }^{v}=0$ for Wave #B) and (iv) both material damping $(\xi =0.02)$ in ${R}_{1}$ and damping of LLR/VLR is considered (${k}_{t,\sigma }^{l}=4.0E7, {c}_{t,\sigma }^{l}=9.90E3$ for Wave #A and ${k}_{t,\sigma }^{v}=1.03E8, {c}_{t,\sigma }^{v}=2.07E4$ for Wave #B). The transmittance ${T}_{\psi }$ of all the four cases for Wave #A is shown in Fig. 8a while Fig. 8b reveals the same trend for Wave #B. As damping of the structure is taken into account, the pass band peaks get lowered while the band gaps broaden and high damping causes the band gaps to vanish.

The effectiveness of the designed LLRs and VLRs is also compared in Fig. 8a,b for both the uncontrolled ${R}_{1}$ (i.e., case 1) against the case when ${R}_{1}$ is attached to LLRs/VLRs (i.e., case iv) with $\sigma =0.20$. In both Fig. 8 and Table 2, it can be observed that a significant reduction in response is achieved for the rail ${R}_{1}$ in the considered frequency range.

**Figure 9:**
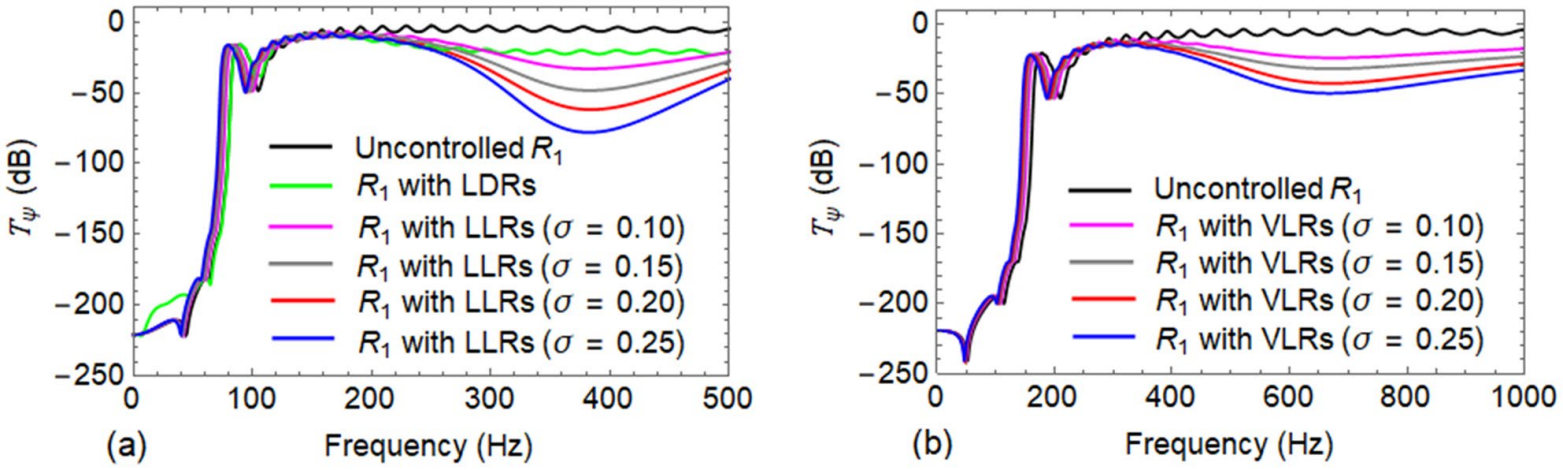
Comparison between transmittance ${T}_{\psi }\left(\mathrm{dB}\right)$ of: (**a**) uncontrolled ${R}_{1}$ against ${R}_{1}$ with LDRs and LLRs in lateral direction; and (**b**) uncontrolled ${R}_{1}$ against ${R}_{1}$ with VLRs in vertical direction.

Similar to the case of LLR, LDR is also designed in the same frequency range. The corresponding optimal stiffness and damping values are obtained as $1.44 \,\mathrm{N}/\mathrm{m}$ and $3.71E4 \,\mathrm{Ns}/\mathrm{m}$, respectively, with the performance metric $\eta =0.147$. For Wave #A, vibration transmittance ${T}_{\psi }$ of uncontrolled ${R}_{1}$ is compared with LDR and LLR of different mass ratio $\sigma$ in Fig. 9a. Similarly, Fig. 9b compares ${T}_{\psi }$ of uncontrolled ${R}_{1}$ against ${R}_{1}$ with VLR for different values of $\sigma$ in case of Wave #B. The efficiency with LDR appears to be less when compared to the LLR. This may be due to low mass mobilization obtained with LDR. In contrast, for a LLR, the complete mass ${m}_{t,\sigma }^{l}$ takes part in the tuned mass damper action; instead, when ${R}_{2}$ is connected to ${R}_{1}$, only a part of the mass of ${R}_{2}$ of a particular span is mobilized, leading to a low effective mass ratio. The LDR also has an additional damping owing to the material property of rail ${R}_{2}$. However, this additional damping cannot offset the effects of the reduced mass mobilization.

### Efficacy of control mechanism

Efficiency of the designed vibration control mechanism in both lateral and vertical direction is evaluated using a general loading scenario. A random Gaussian white noise rotation ${RY}_{i/p}$ and ${RZ}_{i/p}$ about the $y$ and $z$ -axis- is respectively applied for lateral and vertical direction as an input to the left-most end of the rail and the output rotation at the right end is computed as ${RY}_{o/p}$ and ${RZ}_{o/p}$.

**Figure 10:**
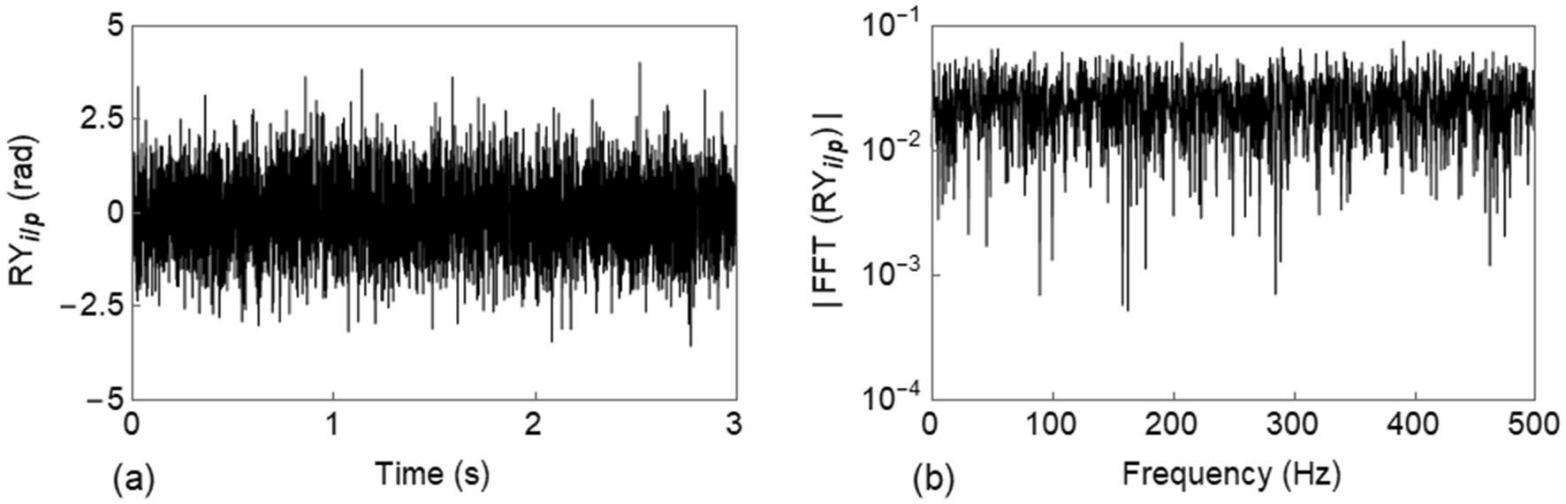
Input rotation (${RY}_{i/p}$) applied to the rail: (**a**) time history; and (**b**) FFT.

The input ${RY}_{i/p}$ is defined as a zero mean Gaussian process with unit standard deviation and is imposed for a duration of $3$ s with a discretization time of $6.25E-4$ s. Figure 10a shows the realization of the input rotation ${RY}_{i/p}$, whilst Fig. 10b depicts the corresponding fast Fourier transform (FFT) which points out the white noise characteristics.

A full transient dynamic analysis is performed by means of the Newmark-β method with the assumption of linear variation of acceleration between two successive time instants^44^ ($\upgamma =1/2$ and $\upbeta =1/6$). Rayleigh damping was provided to the rail and the relevant coefficients were chosen to cover a frequency range of 0 to 500 Hz and 0 to 1000 Hz for lateral and vertical track, respectively. Here, only the LLR/VLR with a mass ratio $\sigma =0.2$ is considered, and since others follow a similar pattern, they are not reported.

**Figure 11:**
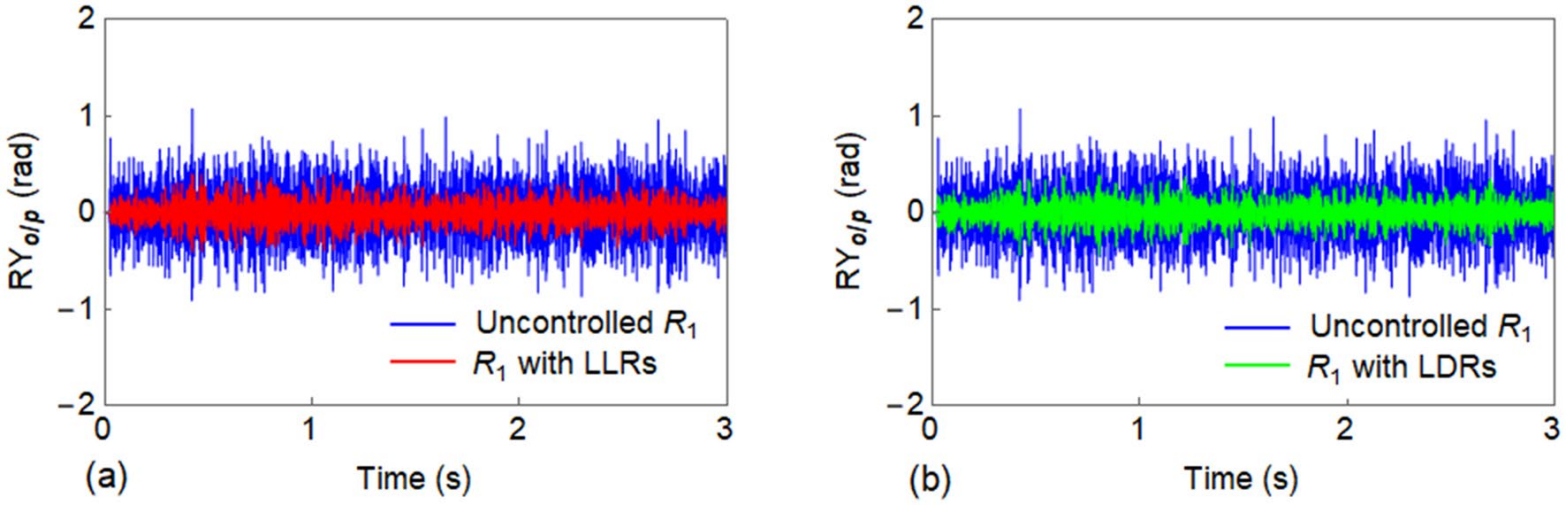
Comparison between the output response (${RY}_{o/p}$) of ${R}_{1}$ in the lateral direction: (**a**) uncontrolled ${R}_{1}$ against ${R}_{1}$ with LLRs; and (**b**) uncontrolled ${R}_{1}$ against ${R}_{1}$ with LDRs.

**Figure 12:**
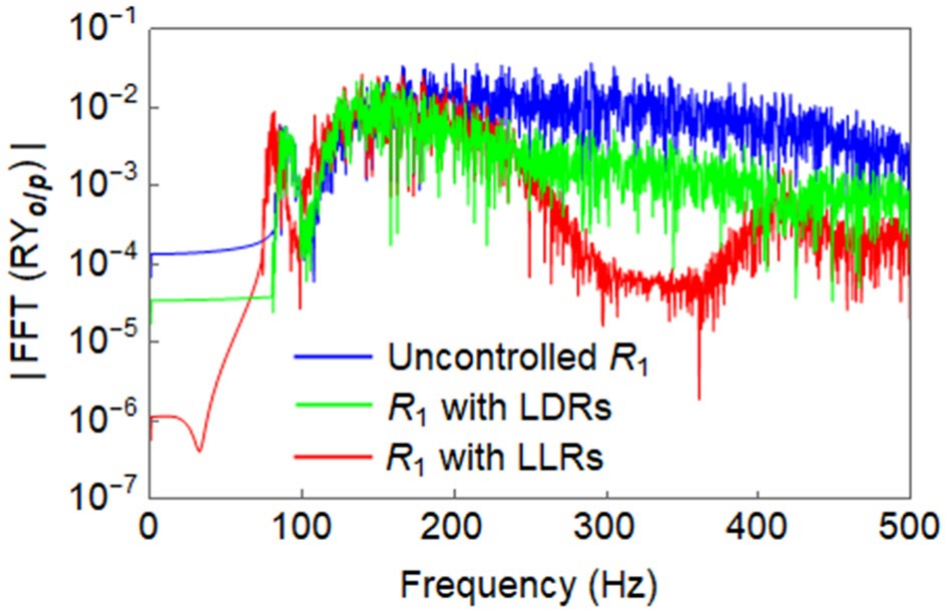
Comparison between the FFT of ${RY}_{o/p}$ of the uncontrolled ${R}_{1}$ against ${R}_{1}$ with LLRs and LDRs in the lateral direction.

The response of uncontrolled ${R}_{1}$ and ${R}_{1}$ with LLRs is compared in Fig. 11a, while Fig. 11b reports the similar comparison for LDRs. A significant reduction in response is observed for both cases. Figure 12 shows the relevant FFT of the responses which further illustrates the response attenuation when properly designed LLRs and LDRs are used.

**Figure 13:**
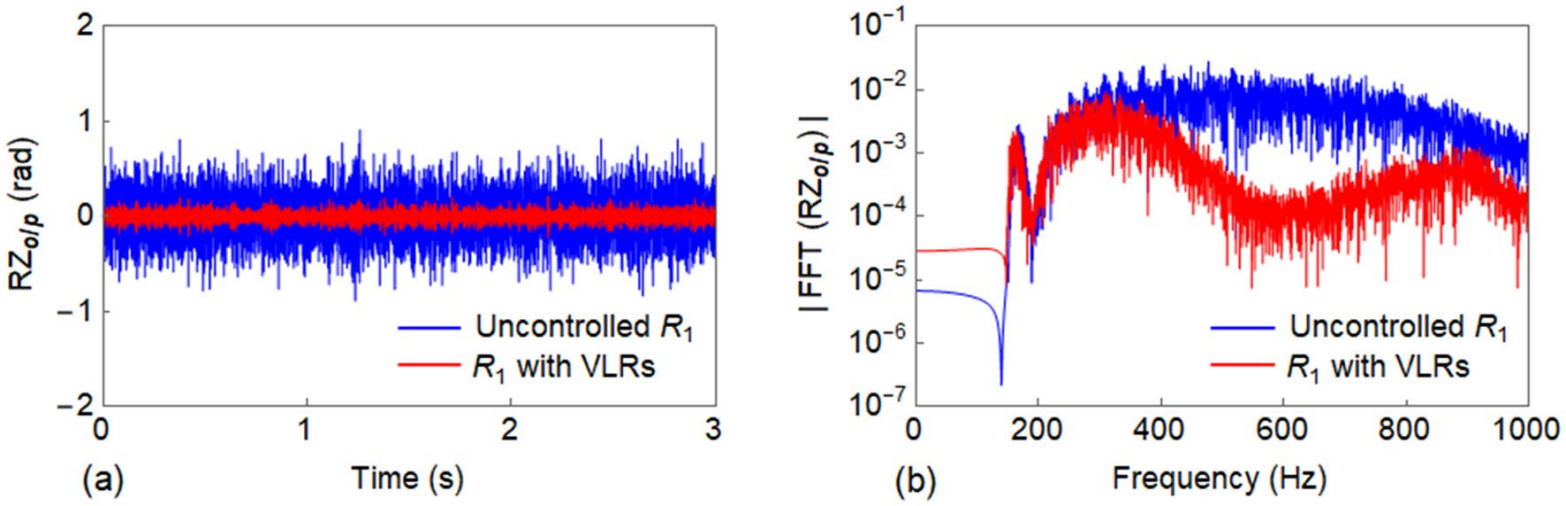
Comparison between the response of uncontrolled ${R}_{1}$ against ${R}_{1}$ with VLRs in vertical direction: (**a**) output response (${RZ}_{o/p}$); and (**b**) FFT (${RZ}_{o/p}$).

Similar to the lateral track, an input rotation ${RZ}_{i/p}$ is applied to the vertical track for $3$ s with a discretization time of $3.25E-4$ s. The response ${RZ}_{o/p}$ of uncontrolled ${R}_{1}$ and ${R}_{1}$ with VLRs is compared in Fig. 13a and their corresponding FFT are represented in Fig. 13b.

## Discussion

To study in depth, the propagation characteristics of flexural wave in rails, a phononic crystal theory-based metamaterial concept was utilized. The dispersion relation for the propagation of both Wave #A and Wave #B in an infinite periodic track was formulated by means of the Floquet-Bloch theorem, and the resulting dispersion characteristics were compared with FE models. Two band gaps were identified for both waves in the considered frequency range. The first band gap is due to local resonance of rail-fastening system while the second one is caused by the spatial periodicity in the track^40^. By virtue of these band gaps, such spatially periodic structures act as filters and allow only waves of particular frequencies to pass through.

Further, to control vibrations within the respective first pass bands of the rail ${R}_{1}$, LLRs/VLRs were attached at the middle of each unit cell of the rail. The elimination of damping of both LLRs/VLRs and the rail ${R}_{1}$, entails a new band gap around the natural frequency of the resonator which can be observed in Fig. 7a,b. Thus, the wave filtering and attenuation capability of the track can be greatly enhanced with the LLRs/VLRs. At the same time, the introduction of resonators causes a small shift in the band gap frequencies as highlighted in Fig. 7. A reader can notice in Fig. 8 that when damping is taken into account in both ${R}_{1}$ and LLRs/VLRs, the vibration transmission peaks are lowered in the pass bands. Although the use of high damping results in vanishing of band gaps ^13,14^, Fig. 8 shows that a significant reduction in the amplitude of vibration^8^ is achieved.

Along these lines, it is apparent from Table 2 that with an increase of the mass ratio $\sigma ,$ the effectiveness of the control mechanism increases in both lateral and vertical directions. However, it also increases the optimal stiffness and damping values which may lead to a higher cost.

Furthermore, to control the vibration of ${R}_{1}$ for the propagation of Wave #A, a strategy based on the novel concept of LDRs was employed. The rail ${R}_{2}$ available in the full track structure was utilized as a LDR. When both the rails ${R}_{1}$ and ${R}_{2}$ are connected by spring-damper systems as in Fig. 2c, ${R}_{2}$ acts as LDR for ${R}_{1}$ and vice versa. Thus, the response of both rails can be equally reduced. From the results of Fig. 12 and the corresponding $\eta$ values of LLRs/LDRs, it is evident that LLRs perform better than the LDRs in terms of response reduction; but the adoption of LDRs may be a cost-saving solution. The vibration reduction principle of the LDR is same as that of the conventional TMD except that in the latter, its complete mass participates in the corresponding mode, while the former being a multi-degrees of freedom (MDoF) system, the full mass mobilization does not occur for any frequency. Also, the LDR has an additional damping due to the material damping of the connected rail. While, a LLR was observed to create stop band around its tuned frequency, the LDR was not capable to do so. This is due to the fact that the LDR is a continuous system. The relevant time history analysis further illustrates the effectiveness of the proposed vibration control mechanisms. Conversely, the corresponding FFT reported in Figs. 12 and 13b demonstrates the efficiency of the designed solution in the considered frequency range for both lateral and vertical track, respectively.

Nonetheless, for any excitation/disturbance whose frequencies fall outside this range, the controller may not exhibit the intended performance. The control strategies of LLR/VLR were based on SDoF resonator systems. This limits their usability for a large frequency range. However, MDoF resonators may efficiently be employed in such situations. While in the context of LDRs, the center of rail ${R}_{1}$ was connected to the center of rail ${R}_{2}$ of the adjacent span, the study of more efficient configurations deserves further attention.
